# Assessment of Vitamin D Status in Slovenian Premenopausal and Postmenopausal Women, Using Total, Free, and Bioavailable 25-Hydroxyvitamin D (25(OH)D)

**DOI:** 10.3390/nu14245349

**Published:** 2022-12-16

**Authors:** Vid Vičič, Andreja Kukec, Saša Kugler, Ksenija Geršak, Joško Osredkar, Ruža Pandel Mikuš

**Affiliations:** 1 Faculty of Health Sciences, Chair of Biomedicine in Healthcare, University of Ljubljana, Zdravstvena pot 5, 1000 Ljubljana, Slovenia; vidvicic@gmail.com; 2 National Institute of Public Health, Trubarjeva 2, 1000 Ljubljana, Slovenia; andreja.kukec@mf.uni-lj.si (A.K.); sasa.kugler@nijz.si (S.K.); 3 Faculty of Medicine, Chair of Public Health, University of Ljubljana, Zaloška 4, 1000 Ljubljana, Slovenia; 4 Faculty of Medicine, Division of Obstetrics and Gynaecology, University of Ljubljana, Šlajmerjeva 3, 1000 Ljubljana, Slovenia; ksenija.gersak@mf.uni-lj.si; 5 Clinic of Gynaecology and Obstetrics, University Medical Centre Ljubljana, Šlajmerjeva 3, 1000 Ljubljana, Slovenia; 6 Institute of Clinical Chemistry and Biochemistry, University Medical Centre Ljubljana, Njegoševa 4, 1000 Ljubljana, Slovenia; josko.osredkar@kclj.si; 7 Faculty of Pharmacy, University of Ljubljana, Aškerčeva 7, 1000 Ljubljana, Slovenia

**Keywords:** vitamin D, 25(OH)D, postmenopausal, premenopausal, bioavailable 25(OH)D, free 25(OH)D, online calculator, cholecalciferol, calcidiol, menopause, DBP

## Abstract

The objective of our study was to evaluate vitamin D status and its predictors in Slovenian premenopausal and postmenopausal women. A cross-sectional study was carried out between 1 March 2021 and 31 May 2021. A total of 319 healthy women from the Central Slovenian region aged between 44 and 65 were recruited; 176 were included in the final analysis. The vitamin D status was determined by measuring the total 25-Hydroxycholecalciferol (25(OH)D) concentration, vitamin D binding protein (DBP), and albumin and calculating the bioavailable 25(OH)D and free 25(OH)D. For the calculation of bioavailable and free 25(OH)D, we developed a new online calculator. The Endocrine Society’s thresholds for vitamin D deficiency and insufficiency were used; 29.0% of premenopausal and 24.4% of postmenopausal subjects were found to be vitamin D deficient (total 25(OH)D < 50 nmol/L); 76.8% of the premenopausal and 61.7% of postmenopausal subjects were found to have insufficient levels (total 25(OH)D < 75 nmol/L). Premenopausal women had 11.8% lower total 25(OH)D, 32.2% lower bioavailable 25(OH)D, and 25.2% higher DBP than postmenopausal women. The most important predictors of vitamin D status were vitamin D supplementation and time spent in the sun. Contrary to similar studies, the vitamin D status in Slovenian postmenopausal women was significantly better than in premenopausal women. In postmenopausal women, the measurement of free or bioavailable 25(OH)D instead of the total 25(OH)D could be advantageous.

## 1. Introduction

**Figure 1:**
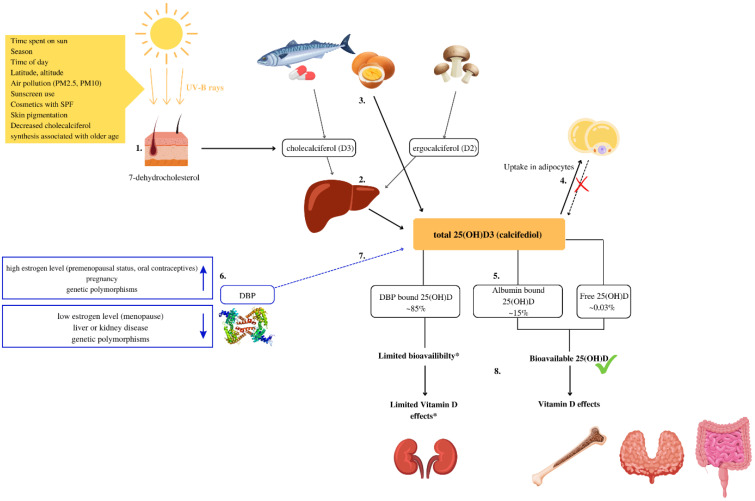
Vitamin D metabolism and bioavailability. (1) Vitamin D is synthesized due to exposure of the skin to UV-B rays from 7-dehidrocholesterol. In the skin, 7-dehydrocholesterol is metabolized into cholecalciferol (D3) [1]. (2) In the liver, cholecalciferol (D3) and ergocalciferol (D2) are metabolized into *25*-Hydroxycholecalciferol (25(OH)D) [1]. (3) Some foods, such as eggs, contain significant amounts of 25(OH)D [2,3]. (4) In obese individuals volumetric dilution of 25(OH)D into the greater volumes of fat, serum, liver, and muscle is probably the most important mechanism that causes lower, serum 25(OH)D in such individuals [4]. (5) In serum, about 0.03% of total 25(OH)D is free, approximately 85% bounds to the vitamin D binding protein (DBP) and 15% to albumin [5]. (6) Several factors can increase or decrease DBP levels and impact 25(OH)D bioavailability [6]. (7) The main biological function of DBP is to extend the plasma half-life of 25(OH)D by decreasing bioavailability [7,8]. (8) Most tissues rely on free and albumin-bound 25(OH)D that are bioavailable and can exert biological activity. * In some cells for example the kidney cells, and likely in the parathyroid gland and placenta cells, DBP-bound vitamin D can be taken up and used [9].

In humans, the main source of Vitamin D is the synthesis of cholecalciferol (D3) from 7-dehydrocholesterol in the skin when exposed to ultraviolet (UV) radiation. To a lesser extent, it is obtained from fatty fish and eggs in the form of cholecalciferol (D3) and in the form of ergosterol (D2) from UV-exposed yeast and fungi [1]. Animal products also contain 25-Hydroxycholecalciferol (25(OH)D) [2], the content of which is rarely included in food composition data [2,3]. The vitamin D metabolism is explained in Figure 1.

In the previous Slovenian study which included 125 adults (18–64 years) and 155 elderly (65–74 years old) subjects, researchers found that in the period between November and April, 81.6% of subjects had vitamin D deficiency (total 25(OH)D < 50 nmol/L) [10].

The decline of estrogens during menopause causes increased bone turnover and decrease in bone mineral density [11], that can be further progressed due to vitamin D deficiency [12]. Furthermore, body composition changes associated with menopause, such as increase in fat mass and decrease in lean muscle mass, [11] also increase the risk of vitamin D deficiency [4]. In older adults decrease in cutaneous cholecalciferol synthesis present a significant risk factor for vitamin D deficiency [13].

Estrogen increases the number of vitamin D receptors in tissues and it is involved in the conversion of 25(OH)D to 1,25(OH)D [14,15]. Women with higher estradiol levels were found to have higher levels of serum 25(OH)D [14,15,16]. In several studies, the observed serum 25 (OH)D in postmenopausal women was lower compared to peri-menopausal women of the same age [16,17].

In serum, about 0.03% of 25(OH)D is free approximately 85% is bound to the vitamin D binding protein (DBP) and 15% to albumin [5]. In accordance with the free hormone hypothesis, the biological activity of the hormone calcitriol depends on its free form (unbound hormone) concentration [18]. Consequently, the bioavailability of serum 25(OH)D of premenopausal and postmenopausal women has to be considered. DBP functions as a vitamin D carrier protein and has a major role in extending the plasma half-life of 25(OH)D by decreasing bioavailability [7,8]. In some cells DBP-bound 25(OH)D is bioavailable. In some kidney cells and likely in the parathyroid gland and placenta cells, DBP participates in the transport of the 25(OH)D into the cell via a megalin/cubilin complex [9].

Most of routinely used vitamin D assays do not distinguish between the DBP-bound 25(OH)D, albumin-bound 25(OH)D and free biologically active vitamin D. Free 25(OH)D can be calculated or analyzed directly [6]. Free 25(OH)D includes only unbound 25(OH)D, whereas bioavailable 25(OH)D comprises free 25(OH)D and albumin-bound 25(OH)D [19]. Liver disease, kidney disease, and conditions such as pregnancy, genetic polymorphisms, and menopause affect DBP or albumin synthesis [6].

The objective of this article is to assess the vitamin D status, its predictors, and differences between premenopausal and postmenopausal women in Slovenia.

## 2. Materials and Methods

### 2.1. Study Design and Participants

A cross-sectional study, HIS (Health Interview Survey) and HES (Clinical Health Examination Survey type) [20] was carried out between 1 March 2021 and 31 May 2021. Three hundred and nineteen (319) healthy women from the Central Slovenian region, aged between 44 and 65, were recruited for the study.

**Figure 2:**
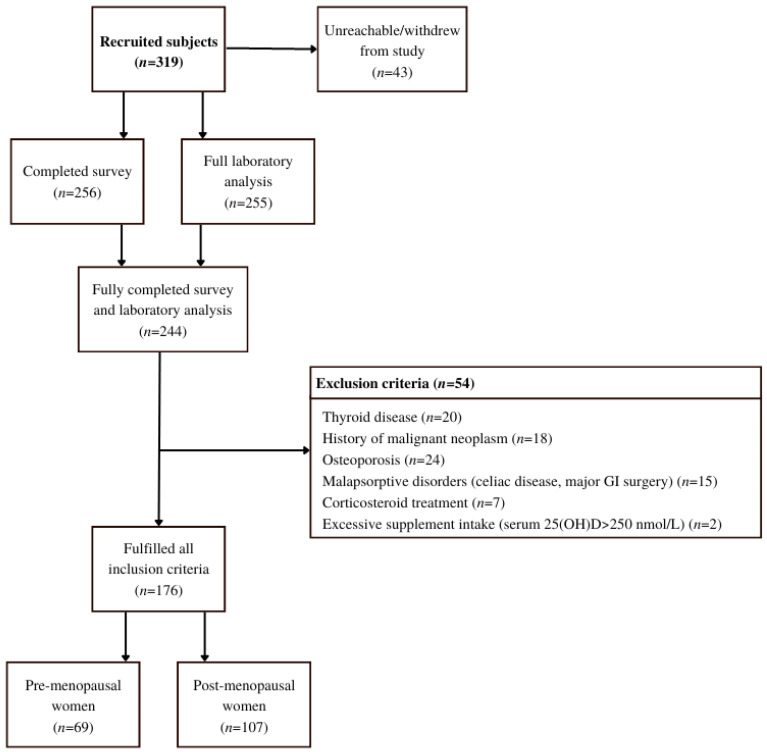
Flow chart of the study that included healthy women from the Central Slovenian region aged between 44 and 65 and was carried out between 1 March 2021 and 31 May 2021.

Participants were initially recruited by healthcare workers in two healthcare centers during their preventive health visits (Figure 2). Since recruitment was hindered by SARS-CoV-2 preventive measures, the snowball sampling method was successfully implemented [21]. Thus, after the initial participants were informed of their vitamin D status, either by post or e-mail, they were asked to invite suitable peers to the study. They were also given a link to an online contact form where new participants could join.

### 2.2. Ethical Approval

The study protocol was approved by the Slovenian National Medical Ethics Committee (Ministry of Health, Republic of Slovenia) under the identification number KME 0120-68/2019/9 (approval letter ID 0120-68/2019/9, date of approval: 22 March 2019). The study was performed in compliance with the requirements of the local authorities. All subjects signed a written consent form (ICF) before participating in the study. The consent form was signed online or in person.

### 2.3. Inclusion and Exclusion Criteria

All women between the age of 44 and 65, willing to participate were included. Menstruation status was determined by questionnaire. WHO definitions of menopause were used. Women were defined as postmenopausal after at least 12 consecutive months of amenorrhea [22].

For the final analysis, the following exclusion criteria which we have summarized and expanded from Brot et al. [23], were considered: (1) confirmed osteoporotic fractures or metabolic bone diseases; (2) current estradiol therapy; prior estradiol therapy is not an exclusion criterion if >3 months have elapsed since the last dose; (3) treatment with corticosteroids in the previous 12 months; (4) treatment of hyperthyroidism or hypothyroidism in the previous 12 months; (5) any new or unregulated chronic disease (e.g., unregulated diabetes); (6) malignant diseases at any time in life; (7) hysterectomy is not an exclusion criterion; (8) health conditions associated with vitamin D deficiency: malabsorption, chronic inflammatory bowel disease, gastrointestinal resection, liver disease, acute gallbladder disease, chronic kidney disease grade 3 to 5; (9) institutionalization and poor physical mobility; (10) excessive vitamin D intake (total 25(OH)D > 250 nmol/L).

### 2.4. Content Validity of a Questionnaire

The first version of the questionnaire was developed and tested in a pilot study conducted in 2019 [24]. Based on the results of the pilot study we created an improved version of the questionnaire. For content validity, a panel of seven experts was used. Item-wise Content Validity Index (CVI) of the experts responses was calculated [25]. S-CVI/Ave (scale-level content validity index based on the average method): 0.96 and S-CVI/UA (scale-level content validity index based on the universal agreement method): 0.81 indexes were obtained.

### 2.5. Data Collection

After obtaining signed consent forms (ICF), a telephone survey was performed by trained registered nurses (RN) or nutritionists (MNutr.) using the specific questionnaire (Supplemental Material).

We formulated the questionnaire based on a modified food frequency questionnaire (FFQ), additionally the questions on body weight, height, health status, use of food supplements with vitamin D, food intake, menstruation/menopause, sun exposure status, skin type, socioeconomic and socio-demographic status were added.

The collection of blood samples was carried out during regular working hours in the chosen healthcare centers and University Medical Centre Ljubljana between 1 March 2021 and 31 May 2021. All samples were transported to a central laboratory at the University Medical Centre in Ljubljana, where they were analyzed the same day or stored at −80 °C until analyzed.

Measurements of total 25(OH)D, albumin, DBP and estradiol were measured in the serum of all the participants, using the following methods: Architect 25-OH D vitamin kit (Abbott Diagnostics, Lake Forest, IL, USA), ADVIA^®^ 1650 Chemistry Albumin BCP Assay (Siemens, New York, NY, USA), Human Vitamin D Binding Protein SimpleStep ELISA^®^ Kit, ab223586 (Abcam, Cambridge, UK), LIAISON^®^ Estradiol II Gen REF: 310680 (DiaSorin, Saluggia, Italy). All measurements were performed at the Clinical Institute of Clinical Chemistry and Biochemistry (University Medical Center, Ljubljana, Slovenia).

### 2.6. Calculation of Bioavailable and Free 25(OH)D

The calculation of free and bioavailable 25(OH)D was based on a modified Vermulen equation [26] with the exact calculation procedure as instructed by Abidin and Mitra (2020) [19], presented in Theorem 1. We prepared a free online calculator application available at: www.vidvicic.com/dcalc (accessed on 10 September 2022).


(1) Bioavalible 25(OH)D=[Free25(OH)D]+[AlbB 25(OH)D]

(2) Bioavailable 25(OH)D = [Free 25(OH)D] + [DAlb] = (Kalb × [Alb] + 1) × Free 25(OH)D

(3) $$\mathrm{Free}25(\mathrm{OH})D\;=\frac{-b+\sqrt{b^{2}-4\mathrm{ac}}}{2a}$$

(4) a = KDBP × Kalb × [Alb] + KDBP

(5) b = (KDBP × [DBP]) − (KDBP × [Total 25(OH)D]) + (Kalb × [Alb]) + 1

(6) c = −[Total 25(OH)D]

(7) [DAlb] = Bioavailable 25(OH)D − Free 25(OH)D

Kalb = affinity constant between 25(OH)D and albumin = 6 × 105 M − 1
KDBP affinity constant between 25(OH)D and DBP 7 × 108 M − 1
[Alb] = concentration of albumin
[Total 25(OH)D] = concentration of total 25(OH)D
[DBP] = concentration of DBP
[DAlb] = Albumin-bound 25(OH)D

**Theorem** **1.** *Equations for calculation of free and bioavailable vitamin D* [19,26].

### 2.7. Statistical Analysis

In the statistical analysis the observed variables were total 25(OH)D, albumin, DBP, estradiol, calculated free 25(OH)D, and calculated bioavailable 25(OH)D. The explanatory variables were supplementation (frequency and quantity) with vitamin D, eating habits—frequency and quantity, vitamin D content, sun exposure), BMI, and menstruation status (premenopause, postmenopause).

Total 25(OH)D levels in serum were assessed using Endocrine Society cut-off values: Deficiency: <50 nmol/L, Insufficiency: 50–75 nmol/L and target concentration for optimal vitamin D effects 75–125 nmol/L [27,28].

The data was normally distributed. Differences between groups were examined with a two independent samples *t*-test. A Pearson correlation coefficient was used to assess the linear association, where we interpreted the results according to Mukaka [29]. Values are presented as mean ± SD or as a percentage (%) in the case of categorical variables. Odds ratios (OR) were calculated using a two-by-two frequency table [30]. For odds ratios, 95% confidence intervals were calculated. The level of significance was set at 𝑝 < 0.05.

The statistical analysis was conducted using MS Excel 2019 and SPSS, version 27 (IBM, 2020).

## 3. Results

### 3.1. Descriptive Statistics

The Endocrine Society’s thresholds for vitamin D deficiency and insufficiency were used, according to which 29.0% of the premenopausal and 24.4% of postmenopausal women were found to be vitamin D deficient. 76.8% of the premenopausal and 61.7% of postmenopausal women were found with insufficient total 25(OH)D levels: <75 nmol/L (Table 1).

### 3.2. Comparison of Premenopausal and Postmenopausal Women

**Figure 3:**
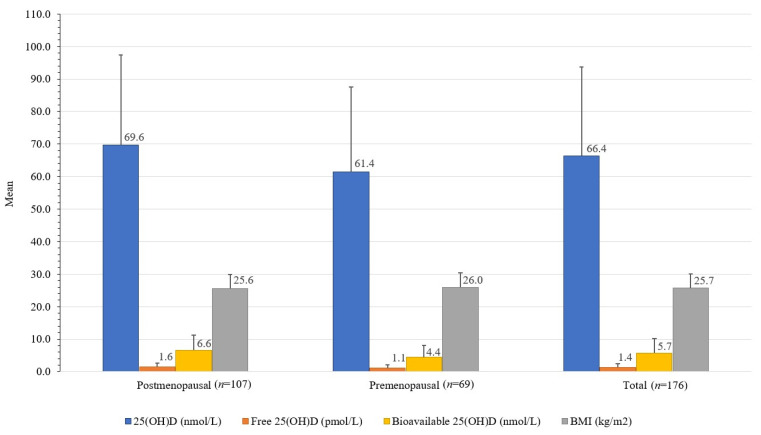
Differences between serum total, free, and bioavailable 25(OH)D concentrations and BMI in healthy women from the Central Slovenia region, aged 44 to 65 years, included in the study carried out between 1 March 2021 and 31 May 2021 (*n* = 176).

Premenopausal women had 32.2% lower bioavailable 25(OH)D (4.4 ± 3.8 nmol/L) than postmenopausal women (6.6 ± 4.7 nmol/L); *t*(173) = [3.22], *p* 0.002, and 11.8% lower total 25(OH)D (61.4 ± 26.1 nmol/L) than postmenopausal women (69.6 ± 27.8 nmol/L); *t*(173) = [8.2], *p* 0.052. Premenopausal women also had 25.2% higher DBP (680 ± 486 mg/L) than postmenopausal women (509 ± 387 mg/L); *t*(174) = [2.596], *p* 0.01 (Table 1, Figure 3).

There was a significant difference in the odds of having vitamin D insufficiency (<75 nmol/L total 25(OH)D) between the premenopausal and postmenopausal women (OR = 2.06, *p* 0.033, 95% CI [1.06, 3.99]). There was a non-significant difference in the odds of having vitamin D deficiency (<50 nmol/L total 25(OH)D) between the premenopausal and postmenopausal women (OR = 1.42, *p* 0.33, 95% CI [0.70, 2.89]).

### 3.3. Correlations of Total, Free, and Bioavailable 25(OH)D

Furthermore, we found a low positive correlation between calculated bioavailable 25(OH)D and total 25(OH)D (r (175) = 0.44, *p* < 0.001), and a low positive correlation between calculated free 25(OH)D and total 25(OH)D (r (175) = 0.42, *p* < 0.001). We found a negligible negative correlation between menopause and DBP (r (175) = −0.16, *p* 0.018) (Table 2).

Supplemental vitamin D intake (r (175) = 0.56, *p* < 0.001) and time spent in the sun (r (175) = 0.23, *p* [0.003]) were found to be a significant predictors of vitamin D status. Food vitamin D intake, physical activity, and BMI were not significant (Table 3).

### 3.4. Predictors of Vitamin D Status

A total of 61.4% of participants were supplementing with at least 5 µg of vitamin D prescribed by physician or as food supplements. There was a significant difference in the odds of having vitamin D deficiency (<50 nmol/L total 25(OH)D) between the non-supplementers and supplementers with >5 µg of vitamin D/day (OR = 7.59, *p* < 0.001, 95% CI [3.46, 16.68]).

There was a significant difference in the odds of having vitamin D insufficiency (<75 nmol/L total 25(OH)D) between the non-supplementers and supplementers with >5 µg of vitamin D/day (OR = 6.23, *p* < 0.001; 95% CI [2.72, 14.274]).

## 4. Discussion

The total 25(OH)D in Slovenian postmenopausal women was significantly higher than in premenopausal women. The difference is even more evident when comparing bioavailable and free 25(OH)D. This may be due to statistically non-significant (*p* = 0.069) higher supplement use in postmenopausal women.

Compared to previous Slovenian studies, the results from our study indicate almost 7-time increase in vitamin D supplementation prevalence compared to Slovenian population in the study done before SARS-CoV-2 pandemic [10].

The question remains if bioavailable 25(OH)D would be a better biomarker related to health outcomes than total 25(OH)D. DBP is associated with estradiol levels and is lower in postmenopausal women [14,15,16,17]. Pop and Shapses [17] found 18% lower concentrations of total 25(OH)D in serum in postmenopausal women compared to premenopausal women, but the calculated bioavailable vitamin D was only 9% lower.

Several studies investigated whether bioavailable 25(OH)D would be better associated with bone mineral density markers [19,31]. One such case is Vitamin D Paradox in African American women having lower total 25(OH)D but half rate of bone fractures compared to white American women. African American women had significantly lower total 25(OH)D than white American women, but bioavailable 25(OH)D was not significantly different [32]. Calculated free 25(OH)D and bioavailable 25(OH)D were found to be more strongly correlated with bone mass density in postmenopausal women than total 25(OH)D [19].

The use of free 25(OH)D may be valuable in liver disease, kidney disease, and conditions such as pregnancy, genetic polymorphisms [6], ethnically diverse populations [6,19,32], and menopause [17,19]. Due to lower DBP, postmenopausal women have higher bioavailability of 25(OH)D [17]. Therefore, the determination of bioavailable 25(OH)D or free 25(OH)D was necessary for a comparison of vitamin D status between premenopausal and postmenopausal women. However, there is indicated the need for a larger study with higher statistical power where reference values should be established [6]. Zeng and others [33] derived reference values for free 25(OH)D using linear regression models but concluded that clinical studies were needed to confirm their suggestions. Any reference values will need to consider the discrepancy between directly measured free 25(OH)D and calculated free 25(OH)D. This discrepancy is larger in African Americans and is contributed to difference in DBP binding ability between ethnic groups [34]. Tsuprykov with colleagues [35] calculated different reference values for pregnant women for both calculated and measured free 25(OH)D and concluded that both methods are suitable.

In our study, supplemental intake of vitamin D and time spent in the sun were significant predictors associated with total 25(OH)D in serum. The association with BMI was not significant due to the low number of obese subjects. Studies with more obese subjects report BMI as a significant risk factor for vitamin D deficiency [36,37,38,39]. As in our study, the effect of dietary vitamin D intake in countries without food fortification is usually not significant [39,40].

### 4.1. Study Strengths and Limitations

The main strength of our study is that it is the first to analyze the status of the total, bioavailable, and free 25(OH)D in a subpopulation of premenopausal and postmenopausal women ins Slovenia. We have included 0.4% of all women aged between 45 and 65 years in the Central Slovenian region. To this date, this is by far the largest vitamin D survey in Slovenia done on sample of premenopausal and postmenopausal women.

However, we cannot generalize the results to the entire population, as the study was performed during a total SARS-CoV-2 lockdown that influenced vitamin D intake and lifestyle. The choice to be included in the study was offered to all health center visitors. Those who chose to be included in this voluntary study had significantly higher education and lower BMI compared to the general population [41]. Additionally, socioeconomic factors, such as language barrier, access to transportation, and work time flexibility, influenced the withdrawal from the study.

### 4.2. Practical Considerations and Future Research

We did not assess bone health and markers of other important diseases to establish if bioavailable/free 25(OH)D better predicts the risk for low bone mass density and other conditions. In future follow-up studies, the levels of vitamin D supplementation should be assessed to determine if the SARS-CoV-2 pandemic inspired vitamin D supplementation persisted.

## 5. Conclusions

Contrary to similar studies, the vitamin D status in Slovenian postmenopausal women was significantly better than in premenopausal women.

In postmenopausal women, the measurement of free or bioavailable 25(OH)D instead of total 25(OH)D may be advantageous. More studies need to be performed to establish reference values.

The most important predictor of vitamin D status was vitamin D supplementation. The high proportion of supplementation with vitamin D could be attributed to increased awareness due to the SARS-CoV-2 pandemic. This is also evident when comparing our results to studies done pre-SARS-CoV-2 pandemic.

## Figures and Tables

**Table 1:** Population characteristics, vitamin D status, supplementation, and food intake of healthy women, aged between 44 and 65, from the Central Slovenian region, who were included in the study carried out between 1 March 2021 and 31 May 2021 (*n* = 176).

Variable	Category/ Unit	Total *n* = 176	Premenopausal *n* = 69	Postmenopausal *n* = 107	𝑝-Value
Age	years	53.8 ± 5.0	49.8 ± 3.3	56.45 ± 4.1	**<0.001**
BMI	kg/m^2^	25.7 ± 4.4	26.0 ± 4.4	25.6 ± 4.3	0.574
	18.5–24.9	51.7%	50.7%	52.3%	
	25.0–29.9	32.4%	34.8%	30.8%	
	30.0–34.9	10.8%	8.7%	12.2%	
	35.0–39.9	4.6%	5.8%	3.7%	
	>40.0	0.57%		0.93%	
Lifestyle Factors					
Smoking status	Current smoker	13.0%	17.4%	10.3%	0.092
	Former smoker	24.4%	17.4%	29.0%	
	Non-smoker	62.5%	65.2%	61.0%	
Education level	Primary and high school	32.4%	24.6%	37.4%	**0.015**
	Higher education	67.6%	75.4%	62.6%	
Time spent in the sun	min	53.3 ± 17.7	52.9 ± 15.0	53.6 ± 19.3	0.650
Moderate physical activity	h/week	3.2 ± 4.2	3.7 ± 5.3	2.8 ± 3.6	0.197
	>150 min/week	86.9%	82.6%	89.7%	
	<150 min/week	13.2%	17.4%	10.3%	
Sunscreen use	Yes	90.9%	85.5%	94.4%	0.184
	No	9.1%	14.5%	5.6%	
Sun tanning habits	High sun exposure	6.8%	10.1%	4.7%	0.161
	Medium sun exposure	64.2%	56.5%	69.2%	
	Low sun exposure	29.0%	33.3%	26.2%	
Laboratory Analysis					
Total 25(OH)D	nmol/L	66.4 ± 27.4	61.4 ± 26.1	69.6 ± 27.8	0.052
	<30	8.5%	11.6%	6.5%	
	30–50	15.9%	17.4%	15.0%	
	50–75	43.2%	47.8%	40.2%	
	>75	32.3%	23.2%	38.3%	
DBP	mg/L	576 ± 436	680 ± 486	509 ± 387	**0.010**
Albumin	g/L	47.1 ± 2.2	46.9 ± 2.3	47.3 ± 2.2	0.245
Free 25(OH)D	pmol/L	1.37 ± 1.06	1.11 ± 0.90	1.56 ± 1.11	**0.005**
Bioavailable 25(OH)D	nmol/L	5.7 ± 4.5	4.4 ± 3.8	6.6 ± 4.7	**0.002**
Estradiol	nmol/L	0.22 ± 0.48	0.41 ± 0.64	0.11 ± 0.30	**<0.001**
Vitamin D Intake and Supplementation					
Food intake	µg/day	2.2 ± 1.3	2.3 ± 1.5	2.1 ± 1.3	0.227
Supplement use (≥5 µg vitamin D/day)		61.4%	53.6%	66.4%	0.069
Supplemental intake	µg/day	21.7 ± 26.2	20.1 ± 28.2	22.8 ± 25.0	0.499
Intake of all sources	µg/day	24.1 ± 26.2	22.4 ± 28.1	25.1 ± 25.0	0.500

BMI = body mass index, DBP = vitamin D binding protein. All values are presented as mean ± SD or %. Values are presented as mean ± SD, *p* < 0.05 is considered statistically significant (*p* values of significant variables are in bold print).

**Table 2:** Pearson’s correlation coefficients (r) of blood biomarkers in healthy women from the Central Slovenian region aged between 44 and 65 years who were included in the study carried out between 1 March 2021 and 31 May 2021 (*n* = 176).

	Total 25(OH)D	Bioavailable 25(OH)D	Free 25(OH)D	Estradiol	DBP	Albumin
Total 25(OH)D	1	0.44 **	0.42 **	−0.14	0.06	0.12
Bioavailable 25(OH)D	0.44 **	1	0.97 **	−0.13	−0.61 **	0.08
Free 25(OH)D	0.42 **	0.97 **	1	−0.13	−0.63 **	0.03
Estradiol	−0.14	−0.13	−0.13	1	0.11	−0.19 *
DBP	0.06	−0.61 **	−0.63 **	0.11	1	0.14
Albumin	0.12	0.08	0.03	−0.19 *	0.14	1

** Correlation is significant at the 0.01 level (2-tailed). * Correlation is significant at the 0.05 level (2-tailed).

**Table 3:** Pearson’s correlation coefficients (r) of predictors of vitamin D status in healthy women from the Central Slovenian region aged between 44 and 65 years, who were included in the study carried out between 1 March 2021 and 31 May 2021 (*n* = 176).

	25(OH)D (nmol/L)
Supplemental vitamin D intake (µg/day)	0.56 *
Food vitamin D intake (µg/day)	0.13
Time spent in the sun (min)	0.23 *
BMI	−0.10
Moderate physical activity h/week	0.06

* Correlation is significant at the 0.05 level (2-tailed).

## Data Availability

The data presented in this study are available on request from the corresponding author.

## Supplementary Materials

The following supporting information can be downloaded at: https://www.mdpi.com/article/10.3390/nu14245349/s1, Survey Questionnaire: Determination of Vitamin D Status in Perimenopausal and Postmenopausal Women.

## Abbreviations

BMI	body mass index
UV	ultraviolet
D3	cholecalciferol
D2	ergocalciferol
Total 25(OH)D	25-Hydroxycholecalciferol, calcifediol, calcidiol, 25-Hydroxyvitamin D3
1,25(OH)D	1,25-dihydroxycholecalciferol
DBP	vitamin D binding protein
SARS-CoV-2	severe acute respiratory syndrome coronavirus 2
FFQ	food frequency questionnaire
HIS	health interview survey
HES	clinical health examination survey
ICF	informed consent form
MNutr	Master of Nutrition
OR	Odds ratio
RN	registered nurse
CVI	Content Validity Index
S-CVI/Ave	scale-level content validity index based on the average method
S-CVI/UA	scale-level content validity index based on the universal agreement method
