# Correction: Vičič et al. Assessment of Vitamin D Status in Slovenian Premenopausal and Postmenopausal Women, Using Total, Free, and Bioavailable 25-Hydroxyvitamin D (25(OH)D). *Nutrients* 2022, *14*, 5349

**DOI:** 10.3390/nu15092103

**Published:** 2023-04-27

**Authors:** Vid Vičič, Andreja Kukec, Saša Kugler, Ksenija Geršak, Joško Osredkar, Ruža Pandel Mikuš

**Affiliations:** 1 Faculty of Health Sciences, Chair of Biomedicine in Healthcare, University of Ljubljana, Zdravstvena pot 5, 1000 Ljubljana, Slovenia; vidvicic@gmail.com; 2 National Institute of Public Health, Trubarjeva 2, 1000 Ljubljana, Slovenia; andreja.kukec@mf.uni-lj.si (A.K.); sasa.kugler@nijz.si (S.K.); 3 Faculty of Medicine, Chair of Public Health, University of Ljubljana, Zaloška 4, 1000 Ljubljana, Slovenia; 4 Faculty of Medicine, Division of Obstetrics and Gynaecology, University of Ljubljana, Šlajmerjeva 3, 1000 Ljubljana, Slovenia; ksenija.gersak@mf.uni-lj.si; 5 Clinic of Gynaecology and Obstetrics, University Medical Centre Ljubljana, Šlajmerjeva 3, 1000 Ljubljana, Slovenia; 6 Institute of Clinical Chemistry and Biochemistry, University Medical Centre Ljubljana, Njegoševa 4, 1000 Ljubljana, Slovenia; josko.osredkar@kclj.si; 7 Faculty of Pharmacy, University of Ljubljana, Aškerčeva 7, 1000 Ljubljana, Slovenia

In the original publication [1], there were two errors, one in Figure 3 and one in Table 1, both concerning the mistakenly placed decimal points.

## Error in Figure and Caption

In the original publication [1], there was a mistake in Figure 3, which shows differences between total 25(OH)D concentrations in serum and BMI in healthy women from the Central Slovenian region aged between 44 and 65, who were included in the study carried out between 1 March 2021 and 31 May 2021 (*n* = 176) as published. During figure editing, the labels for premenopausal and postmenopausal women were accidentally switched. The first group should be postmenopausal and the second group should be premenopausal.

An error in the mean values and SD of free 25(OH)D was also noticed. Due to mistakenly placed decimal points, the values and SD presented in the graph were higher (e.g., instead of 1.37, the value appeared as 13.7). 

Additionally, there was a mistake in the caption of Figure 3. The figure presents differences between total, free, and bioavailable 25(OH)D concentrations. However, in the published version, only total 25(OH)D concentrations are mentioned. For better clarity, the caption has been corrected to include the missing information.

The corrected Figure 3 appears below. 

## Error in Table

In the original publication [1], there was a mistake in Table 1, which shows population characteristics, vitamin D status, supplementation, and food intake of healthy women, aged between 44 and 65, from the Central Slovenian region, who were included in the study carried out between 1 March 2021 and 31 May 2021 (*n* = 176). Mean values and SD of free 25(OH)D were presented with wrong decimal point. The corrected Table 1 appears below.

The authors apologize for any inconvenience caused and state that the scientific conclusions are unaffected. This correction was approved by the Academic Editor. The original publication has also been updated.

## Figures and Tables

**Figure 3:**
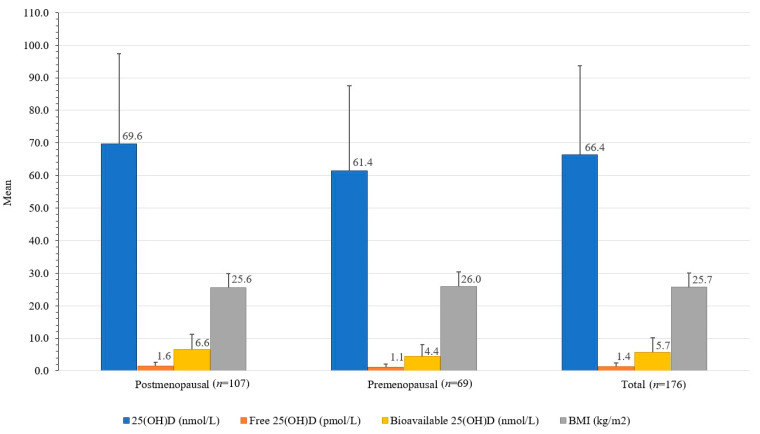
Differences between serum total, free, and bioavailable 25(OH)D concentrations and BMI in healthy women from the Central Slovenia region, aged 44 to 65 years, included in the study carried out between 1 March 2021 and 31 May 2021 (*n* = 176).

**Table 1:** Population characteristics, vitamin D status, supplementation, and food intake of healthy women, aged between 44 and 65, from the Central Slovenian region, who were included in the study carried out between 1 March 2021 and 31 May 2021 (*n* = 176).

Variable	Category/ Unit	Total *n* = 176	Premenopausal *n* = 69	Postmenopausal *n* = 107	𝑝-Value
Age	years	53.8 ± 5.0	49.8 ± 3.3	56.45 ± 4.1	**<0.001**
BMI	kg/m^2^	25.7 ± 4.4	26.0 ± 4.4	25.6 ± 4.3	0.574
	18.5–24.9	51.7%	50.7%	52.3%	
	25.0–29.9	32.4%	34.8%	30.8%	
	30.0–34.9	10.8%	8.7%	12.2%	
	35.0–39.9	4.6%	5.8%	3.7%	
	>40.0	0.57%		0.93%	
Lifestyle Factors					
Smoking status	Current smoker	13.0%	17.4%	10.3%	0.092
	Former smoker	24.4%	17.4%	29.0%	
	Non-smoker	62.5%	65.2%	61.0%	
Education level	Primary and high school	32.4%	24.6%	37.4%	**0.015**
	Higher education	67.6%	75.4%	62.6%	
Time spent in the sun	min	53.3 ± 17.7	52.9 ± 15.0	53.6 ± 19.3	0.650
Moderate physical activity	h/week	3.2 ± 4.2	3.7 ± 5.3	2.8 ± 3.6	0.197
	>150 min/week	86.9%	82.6%	89.7%	
	<150 min/week	13.2%	17.4%	10.3%	
Sunscreen use	Yes	90.9%	85.5%	94.4%	0.184
	No	9.1%	14.5%	5.6%	
Sun tanning habits	High sun exposure	6.8%	10.1%	4.7%	0.161
	Medium sun exposure	64.2%	56.5%	69.2%	
	Low sun exposure	29.0%	33.3%	26.2%	
Laboratory Analysis					
Total 25(OH)D	nmol/L	66.4 ± 27.4	61.4 ± 26.1	69.6 ± 27.8	0.052
	<30	8.5%	11.6%	6.5%	
	30–50	15.9%	17.4%	15.0%	
	50–75	43.2%	47.8%	40.2%	
	>75	32.3%	23.2%	38.3%	
DBP	mg/L	576 ± 436	680 ± 486	509 ± 387	**0.010**
Albumin	g/L	47.1 ± 2.2	46.9 ± 2.3	47.3 ± 2.2	0.245
Free 25(OH)D	pmol/L	1.37 ± 1.06	1.11 ± 0.90	1.56 ± 1.11	**0.005**
Bioavailable 25(OH)D	nmol/L	5.7 ± 4.5	4.4 ± 3.8	6.6 ± 4.7	**0.002**
Estradiol	nmol/L	0.22 ± 0.48	0.41 ± 0.64	0.11 ± 0.30	**<0.001**
Vitamin D Intake and Supplementation					
Food intake	µg/day	2.2 ± 1.3	2.3 ± 1.5	2.1 ± 1.3	0.227
Supplement use (≥5 µg vitamin D/day)		61.4%	53.6%	66.4%	0.069
Supplemental intake	µg/day	21.7 ± 26.2	20.1 ± 28.2	22.8 ± 25.0	0.499
Intake of all sources	µg/day	24.1 ± 26.2	22.4 ± 28.1	25.1 ± 25.0	0.500

BMI = body mass index, DBP = vitamin D binding protein. All values are presented as mean ± SD or %. Values are presented as mean ± SD, *p* < 0.05 is considered statistically significant (*p* values of significant variables are in bold print).
