# Supplementary material for: Assessment of Vitamin D Status in Slovenian Premenopausal and Postmenopausal Women, Using Total, Free, and Bioavailable 25-Hydroxyvitamin D (25(OH)D)
Source: Nutrients. 2022 Dec 16;14(24):5349. doi: 10.3390/nu14245349 (PMC9784017; doi:10.3390/nu14245349)
Supplement: Supplementary file 1 [file nutrients-14-05349-s001.zip › nutrients-2006102-supplementary.pdf]

SURVEY QUESTIONNAIRE:

DETERMINATION OF VITAMIN D STATUS IN PERIMENOPAUSAL AND POSTMENOPAUSAL WOMEN

A – GENERAL

Name		
Surname		
Age		
Body mass		
Body height		
City of residence (no address)		
Phone Number		
E-mail		

B– HEALTH INFORMATION

1.	In the last 12 months, have you been treated for thyroid disease (receiving medication, radioactive iodine)?	
	a) NO<br>b) YES	

2.	Have you ever had cancer?	
	a) NO<br>b) YES	

3.	Have you been diagnosed with osteoporosis?	
	a) NO<br>b) NO, just reduced bone density<br>c) YES	

4.	Do you have any of the following conditions: Crohn's disease, ulcerative colitis, celiac disease, gastric bypass surgery, or chronic kidney disease (stages 3 to 5).	
	a) NO<br>b) YES	

5.	Have you received corticosteroid pills or injections in the last 12 months? Examples: Medrol, Dexamethasone	
	a) NO<br>b) YES	

6.	Have you recovered from COVID19?	
	a) YES, confirmed by test<br>b) YES, I had symptoms of infection, without confirmation with a test<br>c) NO<br>d) Other______________________	

C – INFORMATION ON MENSTRUAL CYCLE

1.	Which statement best describes your **current** menstrual state?	
	a) My periods have become irregular in recent years or months. The time between two periods is now longer or shorter than before, the periods are stronger or weaker than before.<br>b) I have regular periods.<br>c) I'm on birth control.<br>d) I don't have periods anymore, I'm in menopause. Date of my last period: ___/___/____ (at least a year).<br>e) I have been through menopause, however I am currently on hormone replacement therapy. Date of my last period: ___/___/____ (at least a year).<br>f) I had a surgical procedure which caused my periods to stop.<br>g) I've had chemotherapy or radiotherapy which has caused my periods to stop.<br>h) Other: ___________________________________	

2.	How old were you when your menstruation began?<br>_____________years	

3.	What is your menstruation like **currently** (in the last 12 months):	
	a) How many days are there between two menstruations?_________<br>b) How many days does menstruation last?____________<br>Any peculiarities:_____________________________________<br>c) I don't have menstruation	

D - VITAMIN D INFORMATION

1.	Has the 2020 COVID 19 epidemic affected your intake of vitamin D from dietary supplements or medicines?	
	a) I did not take vitamin D supplements or medicines before March 2020, now I do.<br>b) I have taken vitamin D before March 2020 and now I take a higher dose.<br>c) It had no effect.<br>d) I was taking vitamin D before March 2020, but now I don't take it anymore	

2.	Do you take vitamin D supplements?	
	a) NO<br>b) YES<br><br>Product Name: ___________________Dose: _________________<br><br>Product Name: ___________________Dose: _________________	

3.	Do you take medicines with vitamin D, e.g. Plivit D3, Oleovit D3, Vitamin D Krka 1000 IE or another?	
	a) NO<br>b) YES<br><br>Product Name: ___________________Dose: _________________<br><br>Product Name: ___________________Dose: _________________	

4.	Do you take multivitamin supplements or supplements for immunity or any other nutritional supplements?	
	a) NO<br>b) YES<br><br>Product Name: ___________________Dose: _________________<br><br>Product Name: ___________________Dose: _________________	

E – BEHAVIORAL FACTORS

1.	Which statement best describes your skin type?	
	a) The sun burns me very quickly, I never tan. My skin is pale, and possibly freckled. I have red or blonde hair. I have light eye color (green, blue).<br>b) The sun burns me quickly, I tan very little. I have light hair (blonde or light brown). My skin is fair. I have light eye color (green, blue).<br>c) The sun burns me sometimes, but I get an even tan. My skin is fair (when not tanned). I have light or dark eye color.<br>d) The sun rarely burns me, I tan quickly and evenly. My skin is light brown (when not tanned). I have dark eye color.<br>e) The sun rarely burns me, I tan very quickly and evenly. My skin is dark brown. I have dark eye color.<br>f) The sun never burns me, my skin is very dark brown to almost black. I have dark eye color.	

2.	Have you tanned (due to sun exposure) in the last 12 months?	
	a) NO<br>b) YES, all over the body<br>c) YES, especially on exposed areas: face and hands	

3.	Do you use sunscreen?	
	a) NO, never<br>b) YES<br>c) YES, but only during prolonged sun exposure (beach, mountains)	

4.	How much time did you spend outdoors on an average day in the last 7 days?	
	a) less than 5 minutes a day<br>b) 5 to 15 minutes a day<br>c) 15 to 30 minutes a day<br>d) 30 to 60 minutes a day<br>e) 60 minutes or more<br>f) Other:_______	

5.	Have you used a tanning bed in the last 12 months?	
	a) NO<br>b) YES	

6.	How often do you use make-up or SPF cream?	
	a) never<br>b) less than 1 day per week<br>c) 2 to 3 days a week<br>d) 4 to 5 days a week<br>e) 6 to 7 days a week	

7.	Which statement best describes your sun tanning habits?	
	a) I avoid the sun almost completely; I don't tan in the summer, I spend a lot of time indoors.<br><br>b) even in summer I tan only slightly, I regularly use sunscreen .<br><br>c) I don't expose myself to the sun on purpose, but I protect myself when the sun is strong and I'm exposed for a long time. In the summer I tan mostly on exposed areas (face, hands, part of the legs); at the beach or in the mountains I almost always use sunscreen.<br><br>d) I spend a lot of time in the sun. In the summertime I regularly sunbathe and get quite a tan. I work outdoors (e.g. agricultural work). I don't use sunscreen regularly.	

F – NUTRITIONAL FACTORS

The first two questions are about fish. Read the descriptions carefully, as fish species vary greatly in composition. Fill in **only one** (the most appropriate) box.

1.	How often have you eaten sardines, tuna, salmon, mackerel, trout, herring, or other oily fish, in the last 12 months? Ignore lean fish.	

How many times a week					
**1**	**2**	**3**	**4**	**5**	**6**

How many times per month			
**≤1**	**2**	**3**	**4**

2.	How often have you eaten sea bass, sea bream, hake, cod, whiting, common dentex, sole or other lean fish, in the last 12 months?	

How many times a week					
**1**	**2**	**3**	**4**	**5**	**6**

a month			
**≤1**	**2**	**3**	**4**

3.	How often have you eaten eggs in the last 12 months?	
	a) I do not consume eggs or <1 egg per week<br>b) 1 egg per week<br>c) 2 eggs per week<br>d) 3 eggs per week<br>e) 4 eggs per week<br>f) 5 eggs per week<br>g) 6 or more eggs per week. How much? ______	

4.	How often have you consumed milk and liquid dairy products (yogurt, kefir, sour milk) in the last 12 months? Also consider milk in coffee and dishes. Fill in **only one** of the boxes and mark the amount you drink on this occasion.	

How many times a day			
**≤1**	**2**	**3**	**4**

How many times a week					
**1**	**2**	**3**	**4**	**5**	**6**

How many times a month			
**1**	**2**	**3**	**≥4**

How much milk do you drink at once, on average?

a) approx. 0.5 dl
b) 1 dl (half cup)
c) 1.5 dl
d) 2 dl (cup)
e) 3 dl
f) 4 dl
g) more:_______dl

5.	How often have you eaten cheese or cottage cheese in the last 12 months? Fill in **only one** of the boxes and mark the amount you consume on this occasion.	

How many times a day			
**≤1**	**2**	**3**	**4**

How many times a week					
**1**	**2**	**3**	**4**	**5**	**6**

How many times a month			
**1**	**2**	**3**	**≥4**

How much cheese and cottage cheese do you eat at once, on average?

a) 15 g
b) 30 g
c) 45 g
d) 60 g
e) 75 g
f) 90 g
g) more,_______ g

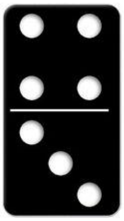

Keep in mind that the weight of a domino-sized slice of cheese is about 30 g.

G – LIFESTYLE FACTORS AND OTHER

1.	What is your highest level of education?	
	a) incomplete primary education<br>b) primary education<br>c) high school<br>d) higher vocational college<br>e) bachelor's or equivalent<br>f) master's or equivalent<br>g) specialization, doctorate	

2.	What social group (or class) do you belong to?	
	a) lower class<br>b) working class<br>c) middle class<br>d) upper middle class<br>e) upper class<br>f) I do not know	

3.	Do you currently smoke or have you ever smoked (cigarettes, cigars or pipe tobacco)?	
	a) I don't smoke and have never smoked<br>b) I smoke now<br>c) I don't smoke now, but I used to smoke	

4.	How physically active are you at work?	
	a) I am not employed (retired, housewife, unemployed)<br>b) I sit most of the time (office work)<br>c) I spend most of my time standing, but the work is not very physically demanding (e.g. saleswoman, hairdresser, security, laboratory technician)<br>d) My job is physically demanding and involves lifting heavy objects, using tools and machinery (e.g. nurse, gardener, assembly line worker)	

5.	In the last 12 months, how many hours per week did you spend on the following activities?		
a)	Planned physical activity: swimming, running, aerobics, fitness, tennis, etc.	_____hours,<br>_____minutes	
b)	Cycling	_____hours,<br>_____minutes	
c)	Walking	_____hours,<br>_____minutes	
d)	Housework, childcare	_____hours,<br>_____minutes	

We sincerely thank you for your cooperation!
